# ClinicalCodes: An Online Clinical Codes Repository to Improve the Validity and Reproducibility of Research Using Electronic Medical Records

**DOI:** 10.1371/journal.pone.0099825

**Published:** 2014-06-18

**Authors:** David A. Springate, Evangelos Kontopantelis, Darren M. Ashcroft, Ivan Olier, Rosa Parisi, Edmore Chamapiwa, David Reeves

**Affiliations:** 1 Centre for Primary Care, Institute for Population Health, University of Manchester, Manchester, United Kingdom; 2 Centre for Biostatistics, Institute for Population Health, University of Manchester, Manchester, United Kingdom; 3 Centre for Health Informatics, Institute for Population Health, University of Manchester, Manchester, United Kingdom; 4 Centre for Pharmacoepidemiology and Drug Safety Research, Manchester Pharmacy School, University of Manchester, Manchester, United Kingdom; 5 Manchester Institute for Biotechnology, University of Manchester, Manchester, United Kingdom; UCL, United Kingdom

## Abstract

Lists of clinical codes are the foundation for research undertaken using electronic medical records (EMRs). If clinical code lists are not available, reviewers are unable to determine the validity of research, full study replication is impossible, researchers are unable to make effective comparisons between studies, and the construction of new code lists is subject to much duplication of effort. Despite this, the publication of clinical codes is rarely if ever a requirement for obtaining grants, validating protocols, or publishing research. In a representative sample of 450 EMR primary research articles indexed on PubMed, we found that only 19 (5.1%) were accompanied by a full set of published clinical codes and 32 (8.6%) stated that code lists were available on request. To help address these problems, we have built an online repository where researchers using EMRs can upload and download lists of clinical codes. The repository will enable clinical researchers to better validate EMR studies, build on previous code lists and compare disease definitions across studies. It will also assist health informaticians in replicating database studies, tracking changes in disease definitions or clinical coding practice through time and sharing clinical code information across platforms and data sources as research objects.

## Introduction

**Figure 1:**
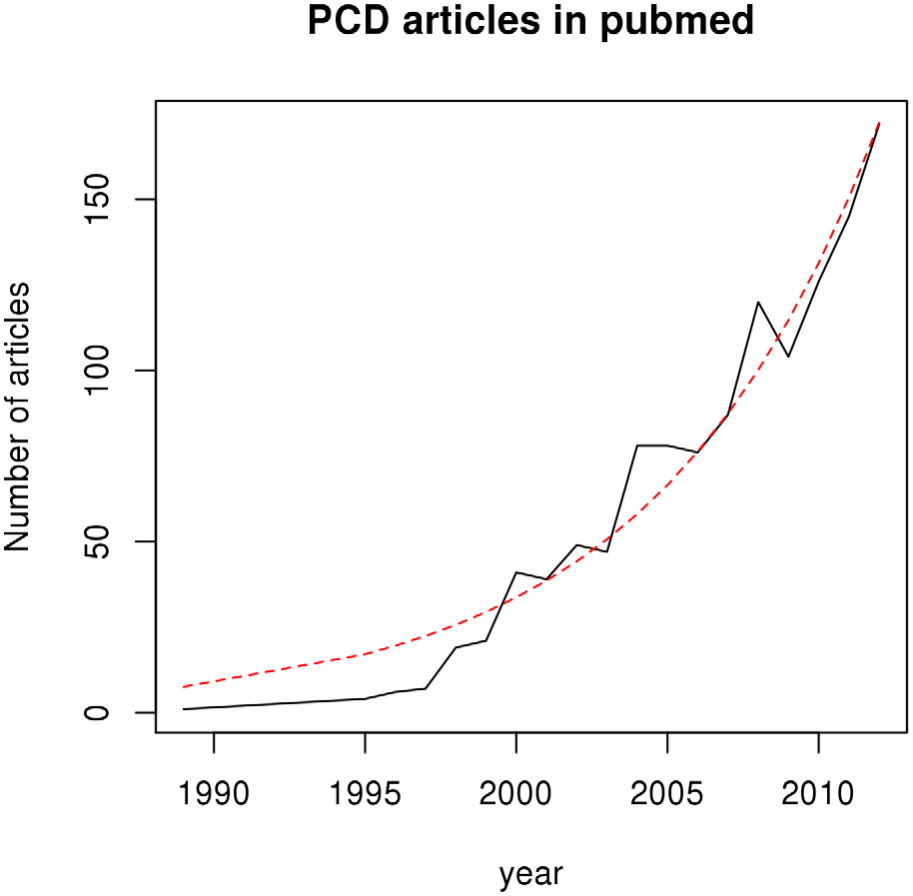
Number of UK Primary Care Database publications.

Over the last 20 years, increasing numbers of general practitioners have used computers to store patients medical records for various administrative functions [1]. Hospitals are also beginning to store their records electronically, though electronic records are far less prevalent than in primary care [2]. These electronic medical records (EMRs) offer great potential for research, enabling the rapid identification of patients for inclusion in intervention and observational studies. As their use becomes more widespread, it is becoming increasingly important to have better means for ensuring and evaluating the validity of studies based on EMRs. EMRs are being used by researchers to address important questions in healthcare that would be difficult or impossible to address using randomised controlled trials, because of the costs involved, the low prevalence of conditions or because a condition may occur in a subgroup such as children or pregnant women. In UK primary care in particular, the annual number of research outputs based on the three main UK primary care databases (The Clinical Practice Research Datalink (CPRD, formerly the General Practice Research Database, GPRD), The Health Improvement Network (THIN) and QResearch) appears to be increasing at an exponential rate (figure 1).

Much research has been done into establishing the internal and external validity of EMR studies [3], particularly from the point of view of data quality, data completeness and confounding. The validity of morbidity registers has also received much attention, through comparison with other sources [4]. There has also been some work replicating studies from one EMR database in another to assess their external validity [5]–[7]. Notwithstanding all of these efforts to establish general validity, the utility of EMR studies has been hampered by poor quality of reporting of research methods and data [8]. One particular area of poor reporting quality is that of clinical coding. Most EMR studies adopt bespoke definitions of clinical entities (such as disease conditions, treatments and diagnostic tests) that are seldom questioned or challenged. These clinical entities are defined through lists of ‘clinical codes’ and the process of preparing these code lists is rarely straightforward and often lacks rigor [9]. Despite calls for greater transparency, sharing of code lists and also for greater use of sensitivity analyses using different sets of codes [10], [11], code lists are still seldom reported in published papers [3]. There is also currently no obligation from funding bodies, journals or regulators for researchers to publish their code lists. Furthermore, there is no centralised repository to hold lists of clinical codes. Consequently, it is impossible to assess the validity of the vast majority of code lists used in EMR research.

There has been a gradual movement towards greater transparency and openness in academic research in recent years [12]–[14], sometimes driven by learned societies [15], and particularly in disciplines where there is high computational load. Furthermore, there is growing pressure from governmental organisations to share and open access to publicly funded research data [16], [17]. In EMR research in particular, there is a movement towards greater transparency and openness in reporting from initiatives such as STROBE [18] and RECORD [8].

To facilitate the transition towards full transparency, we developed www.ClinicalCodes.org, a web repository for EMR researchers to freely upload and download clinical code lists. Below we give an overview of the role and use of clinical codes in EMR research and provide details on the features of the ClinicalCodes repository.

## The role of clinical codes in EMR databases and research

Clinical entities in EMR databases are entered by medical professionals as clinical codes. In UK primary care, Read codes (named after Dr James Read) are the most commonly used, while the ICD-9/10 system (adopted by the World Health Organisation) is more popular in UK hospital settings and primary care in North America and mainland Europe. These codes form a hierarchical classification system for reporting and research purposes and are the essential ‘building blocks’ used to define symptoms, signs and diagnoses, referrals to hospitals and clinics, immunisations, prescribed medications and diagnostic test results.

The process of drawing up code lists to identify clinical entities of interest (e.g patients with a given clinical condition, patients on particular medications, patients with certain diagnostic test, smoking status etc.) is a critical step in setting up EMR studies and multiple code lists will often be required within one study to define multiple conditions, covariates, confounders and outcomes. This is often a complicated and time-consuming process that involves defining the clinical entity of interest and iteratively searching for codes in lookup tables, running searches for codes in different sections of the database, collating the results and classifying them (generally by clinically trained investigators) [9], [19].

The built in flexibility and redundancy of clinical coding systems allows practitioners to use a variety of codes to describe a given condition and minimises their time spent searching for codes, but it presents a challenge to researchers using these codes to effectively define a clinical entity. This flexibility facilitates the clinical use of these codes and minimises the time spent searching for codes by practitioners. However, the multitude of codes for a given condition can present a challenge when data need to be aggregated. For example, the definition of a particular disease condition could include a combination of codes representing diagnoses, symptoms, prescribed drugs and diagnostic tests in order to accurately identify all patients with a certain complicated condition. On the other hand, some entities can be identified with a very simple code list, or even a single clinical code [20].

In any particular application, the set of codes used to define the relevant clinical entities will vary according to the particular question being asked. In some instances it is more important to be all-inclusive and use a broad definition so as not to miss any potential cases; but at other times a narrower definition may be required to focus on cases where diagnosis is more certain. Precisely how a code list is specified can have a major impact on the results of a study [21]. For example, a sevenfold variation in estimates of incidence of rheumatoid arthritis can be largely explained by differences in code-lists between different studies [22], [23]. To account for such variation some studies have used different subsets of code-lists in sensitivity analyses [3], [24]. Furthermore, and in particular for uncommon diseases, small errors in code selection can result in large numbers of misclassified patients, leading to biased results and classification errors affecting conclusions in unpredictable ways [25]. Clinical definitions may also change over time, resulting in a need to revise the corresponding code list [10], a good example being a change in the UK Quality and Outcomes Framework (QOF) Business Rules in 2006. When QOF was first introduced, people with diabetes were identified on the basis of any diabetes code, including non-specific diabetic type codes. From April 2006, the case definition for diabetes was restricted to include only those codes that specified type I or type II diabetes [26]. In practice this meant that about 170 previously used Read codes were no longer being used to identify the condition, a fact that highlights why researchers often need to use a more inclusive (not limited to QOF) code list in order for their research to be robust in the presence of such, more often than not unknown, changes [27]. Finally, different researchers may have different interpretations of the relevance of particular codes.

## Reporting of codes in the current literature

A large component of total EMR research is made up by primary care database (PCD) studies and UK PCDs are among the most researched in the world. Figure 1 shows that research outputs with UK PCDs appear to be increasing at an exponential rate. As one of the largest and most important resources for EMR-based research, it seems reasonable to expect reporting of code lists in UK PCD-based studies to be at least as comprehensive as in other EMR studies. To evaluate levels of transparency in the reporting of clinical code lists, we took a representative sample of UK PCD studies and assessed each study on its extent of reporting of the clinical codes used.

We took a sample of 450 papers from the original 1359 identified from a PubMed search. Of these, 374 (83%) had both the full text accessible to the University of Manchester library and were examples of primary PCD research. Only 5.1% (19 of 374) studies published the entire set of clinical codes needed to reproduce the study (usually in an online appendix), while only an additional 8.6% (32 of 374) stated explicitly that the clinical codes were available upon request (table 1). In a subset of articles published since 2008, 6.9% (16 of 231) published the entire set of codes and 10.4% (24 of 231) stated that clinical codes were available upon request. A breakdown of article numbers, articles with full sets of code lists and articles with codes available on request by year is shown in table 2.

**Table 1:** Percentages of a random sample of UK primary care database studies with details of code lists.

	Number of articles	Percentage
All UK PCD articles	1359	–
In random sample	450	–
Full-text available	417	–
Primary PCD research	374	100
Any code in methods	102	27.2
Any code list in study	60	16
All relevant code-lists	19	5.1
Any codes in paper	102	27.3
Codes available on request	32	8.6
Any codes or available	124	33.2

Percentages are relative to the number of primary PCD research studies.

**Table 2:** Distribution per year of the number of papers using PCDs with full sets of code lists available or codes available on request in a random sample of 374 papers in a PubMed search.

Year	Articles	with all code lists (%)	CAOR* (%)
1996–1997	3	0 (0)	0 (0)
1998–1999	9	0 (0)	0 (0)
2000–2001	21	0 (0)	0 (0)
2002–2003	23	1 (4)	1 (4)
2004–2005	52	2 (4)	3 (6)
2006–2007	35	0 (0)	4 (11)
2008–2009	63	3 (5)	4 (6)
2010–2011	78	7 (9)	9 (12)
2012–2013	90	6 (7)	11 (12)
Total	374	19 (5)	32 (9)

*Code lists stated to be available on request.

## The need for transparency in clinical code usage

We identify four main consequences of lack of transparency of clinical code lists:

1. If code lists are not made available or not published alongside the primary research using them, they represent an important part of a study methodology that is not subject to scrutiny or peer review. In the extreme case, there is no way of assessing the validity of the diagnosis definition used in a study and clinical decisions could be based on invalid results derived from an incorrect patient base. This could happen despite rigorous downstream statistical analysis.
2. The effective replication of EMR studies is dependent on the availability of the clinical codes from the original study. If all of the codes are not available, it is impossible to tell if differences found in study replications are due to artifactual differences in code lists or if they are genuine.
3. If code lists are unknown, comparisons between studies addressing the same clinical question are potentially invalidated. Condition definitions change over time and GP coding practice may also change with respect to regulations and incentives [26]. Also, different studies may use different types of codes for a condition; some studies, for example, include medication and monitoring codes as part of their definition of a patient with diabetes (e.g. [28]) while others do not (e.g. [29]). Not having access to code lists means that it is difficult to know whether fair comparisons are being made between studies.
4. Building code lists is a time consuming process; lack of access to historical code lists means that new lists cannot be built incrementally and iteratively, leading to much ‘reinvention of the wheel’ while decreasing consistency, and potentially accuracy, of definitions across studies.

Although it is now possible to publish clinical codes alongside the original article in an online appendix, keeping lists in this way is difficult to efficiently archive, not readily machine readable and means that codes are kept in an inconsistent manner.

From our study, more studies report that code lists are “available on request” than provide the full code lists as an appendix. This could prove problematic for access at later dates as the researchers may move positions or not respond to requests, rendering the data unavailable.

## The ClinicalCodes online repository

**Figure 2:**
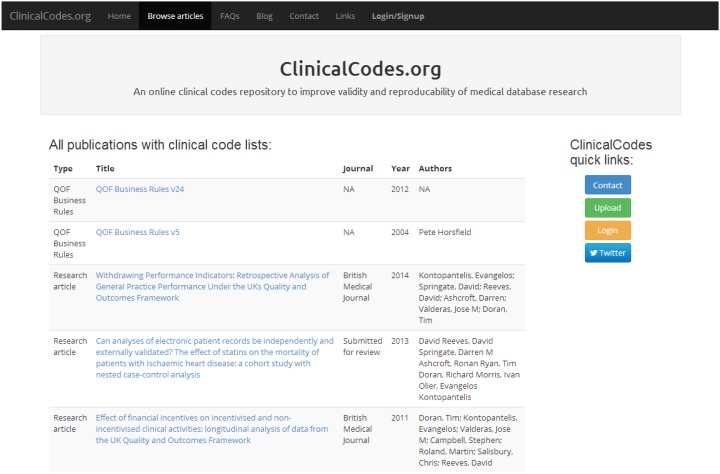
Screenshot of the ClinicalCodes website showing articles with uploaded code lists.

The main ClinicalCodes database consists of a set of ‘Articles’, for each of which a code list, or a collection of code lists, has been uploaded onto the ClinicalCodes.org site. These articles may be, for example, peer-reviewed papers published in medical journals, or other important sources of code lists such as the QOF Business Rule sets (figure 2). Alongside each article is included metadata such as an abstract, citation details, a contact name where possible, and in the case of journal papers, a link to the full text article and DOI. For each article, the associated code lists are detailed and within these the individual clinical codes making up the list. All individual clinical codes are assigned a code name, coding system (Read, OXMIS, SNOMED, CPRD product/medical code, BNF code, OXMIS, ICD-9, ICD-10), description and entity type (diagnostic, drug, test, clinical sign, administrative, demographic, observation, immunisation). Users are able to upload supplementary fields for individual codes or add comments at the code list or article level. Code lists can be downloaded by any user but an account must be created to upload article metadata or code lists or to leave comments. Code lists can be downloaded individually as csv files. If a code lists from a previous article has been used verbatim in a new study, the ClinicalCodes entry for the new study can link to the previous code list. This reduces workload in uploading lists that are unchanged from previous studies while retaining information on the origin of code lists. At the time of submission, the complete code lists used for three papers from our group [7], [24], [29] as well as codes from the UK Quality and Outcomes Framework Business rules versions 5 and 24 have been made available on the repository - a total of 15193 clinical codes across 105 code lists covering medical conditions, lifestyle variables (such as smoking status) physical observations (such as BMI) and testing (for example for retinal screening and blood sugar levels).

We have endeavored to make the upload and download processes as straightforward as possible. In particular, download of individual code lists is a one-click operation requiring no log in or provision of user information. The comments feature, which is available for articles and code lists, enables the study authors to add extra methodological information and also allows other researchers to raise questions and make observations on the code lists which could further assist the development of future code lists.

The website has been extensively tested and is robust enough to function with only very minimal maintenance and the authors have also secured funding to further develop the site, adding more functionality, so the permanence of the project from a technical standpoint is assured.

We have also developed an open-source R package [30] to automate the downloading and importing of clinical code lists from the repository website.

### Clinical code lists as research objects

Research objects are annotated aggregations of data often associated with a scientific publication that facilitate reuse and reproducibility of scientific research [31]. Following this model, metadata and links to code lists for articles are available as research objects that can be shared across platforms in machine readable form. In practice, this means that a JSON (Javascript Object Notation) research object file is available for each article containing: Article metadata (title, author, abstract, reference, link, doi), article level comments, code list level comments and links to the individual code list files. These research object files are available directly by adding a ‘/ro’ to the URI for an article (e.g. www.clinicalcodes.org/medcodes/article/5/ro). The research object format is designed to be available without getting in the way of the main method of download that will be required by most users. The rClinicalCodes R package [30] enables the automated download of code lists and metadata via the research object file. As an example, the JSON research object file for one of the papers in the repository [29] is available at http://dx.doi.org/10.6084/m9.figshare.1008900.

## Discussion

Large electronic medical datasets, including patient medical records databases are already playing an important role in clinical research and this role is set to grow in the era of big data in healthcare [32]. The successful exploitation of large healthcare datasets will depend on the ability of researchers to access and validate data and combine them with other sources [33]. We have developed a repository for clinical codes that will be of great use to two groups of researchers: First, clinical researchers using primary care and other medical databases will be able to more effectively validate their research, build upon previous code lists and match appropriate disease definitions through time. Second, health informaticians will more easily be able to produce study replications (e.g. replications across databases such as [7]), share clinical code data as research objects across platforms and data sources and use the ClinicalCodes database as a research resource in its own right (e.g. to track changes in disease definitions and clinical coding practice through time).

The article classification data suggest that researchers are increasingly making their clinical code lists available in recent years (table 1) but the numbers of researchers doing this are still small and the large majority of new EMR papers still lack this important information.

Researchers using the ClinicalCodes repository can benefit from faster and more consistent development of new code lists, improvements in research quality associated with better scrutiny of lists of clinical codes, greater exposure and potential for studies with uploaded codes to have greater visibility and impact and also from discovering other researchers working in the same area.

Despite these motivations, the success of this project will depend on its widespread adoption by the electronic medical records research community. Although ClinicalCodes solves the problem of having a centralised repository for holding codes, the problem remains that there are few, if any, requirements for researchers to make clinical code lists accessible. We believe that adoption and support of a centralised clinical codes repository by regulators, initiatives such as STROBE and RECORD, funding bodies and publishers of electronic medical records research will be of great benefit to the electronic medical records research community. Clinical codes form an important part of the methods section (i.e. the study results depend on them so they are not ‘data’ as such) of a study and should always be available for critique with the rest of the methods. However, there may be barriers to uptake because of issues around ownership and intellectual property: Researchers may have spent considerable time developing code lists for a study and so may be reluctant to share them without a guarantee of being credited for their work. We would encourage all researchers to appropriately acknowledge reference work on which their own research depends and the clinicalcodes repository facilitates this with the ability to link to code lists from earlier papers. However, there is no mechanism to enforce citation of code lists and researchers are expected to properly cite clinical definitions in the same way that they would be expected to cite other work.

Having openly available code lists will not in itself completely ensure reproducibility of EMR studies. A clinical definition for a complex covariate such as body mass index or smoking status will depend on not only the appropriate code list but a complex algorithm pulling together and processing data from several parts of a database. Although the clinicalcodes repository provides a comment facility which could be used for example program code snippets or algorithm details, full and efficient reproducibility may only be achieved if it becomes common practice for researchers to publish the computer code used in their analysis [34].

A repository for clinical codes is not a panacea for reducing effort in defining clinical entities. There may be a risk that an open clinical codes repository might encourage inertia on the part of researchers by allowing them to simply download existing code lists and rapidly produce research using inappropriate or poorly considered definitions. The current system (or lack of one) should at least mean that code lists are generally developed from scratch on a study-by-study basis, which (although there is some redundancy in this approach) means that researchers are forced to go through the process of carefully considering the appropriate definitions for the study in hand. While this may be the case, the fact that code lists are openly available for critique would mean that studies with poorly considered definitions at least have the possibility of being challenged in the process of post-publication peer review. In addition, it is possible that this kind of inertia in code list choice already exists but with a smaller pool of code lists (within a single research group) and without the possibility of being detected by peers. This repository is a tool and, like all tools (e.g. statistical analysis methods), it can be misused. However, the key issue is transparency and this should inevitably lead to better processes and outputs. We suggest using ClinicalCodes not as a way of short-circuiting effort in developing new definitions, but rather to better employ the scientific method by iteratively building on previous code list research.

### Availability

ClinicalCodes is freely accessable at http://www.clinicalcodes.org. The article classification data is available at http://dx.doi.org/10.6084/m9.figshare.1008899.

## Materials and Methods

### Article Classification

To get an estimate of the extent of the problem of lack of transparency in clinical code-lists in EMR studies, we collected articles conducting primary research using the three major UK-wide Primary care databases (PCDs) (The Clinical Practice Research Datalink (CPRD), formerly the General Practice Research Database (GPRD)); The Health Improvement Network (THIN); QResearch). A Search was made on Pubmed for articles containing any of the following terms in the title or abstract: “CPRD”, “Clinical Practice Research Datalink”, “GPRD”, “General Practice Research Database”, “The Health improvement Network”, “QResearch” up until September 2013, returning 1359 articles. A random sample of 450 articles from this 1359 was taken for further analysis. From this sample, all articles were identified that were both primary EMR research and had their full text accessable via the University of Manchester library (374 articles). We then scored each paper as belonging or not to each of the following categories:

1. Any clinical codes listed in the methods section
2. At least one full code list provided in the paper or in an appendix
3. All code lists provided to enable replication of the study
4. States that “Code lists are available on request”

Analyses were performed using R v2.15.2 [35]. Article counts over time were aggregated using the R package rpubmed (https://github.com/rOpenHealth/rpubmed).

### Database Architecture and Web Interface

The repository data is stored in a relational database called PostgreSQL (http://www.postgresql.org). Server-side web programming was done in Python v2.7.5 (http://www.python.org) using the Django v1.5 web framework (https://www.djangoproject.com). The client side scripting was done in JavaScript and HTML5 and used Twitter Bootstrap v3 (http://getbootstrap.com) as a front-end framework. The dynamic parts of the site were served using Gunicorn v18.0 (http://gunicorn.org) and static parts with Nginx v1.0.15 (http://nginx.org). Cacheing and sessions are handled by a Redis v2.4.10 NoSQL database (http://redis.io). The repository is hosted on a 64 bit Red Hat Enterprise Linux server release 6.4 virtual machine at the University of Manchester.
